# *In-vivo* Sino-Atrial Node Mapping in Children and Adults With Congenital Heart Disease

**DOI:** 10.3389/fped.2022.896825

**Published:** 2022-07-01

**Authors:** Rohit K. Kharbanda, Mathijs S. van Schie, Nawin L. Ramdat Misier, Fons J. Wesselius, Roxanne D. Zwijnenburg, Wouter J. van Leeuwen, Pieter C. van de Woestijne, Peter L. de Jong, Ad J. J. C. Bogers, Yannick J. H. J. Taverne, Natasja M. S. de Groot

**Affiliations:** ^1^Department of Cardiology, Erasmus Medical Centre, Rotterdam, Netherlands; ^2^Department of Cardiothoracic Surgery, Erasmus Medical Centre, Rotterdam, Netherlands

**Keywords:** sino-atrial node, epicardial mapping, congenital heart disease, sinus node dysfunction (SND), atrial fibrillation

## Abstract

**Background:**

Sinus node dysfunction (SND) and atrial tachyarrhythmias frequently co-exist in the aging patient with congenital heart disease (CHD), even after surgical correction early in life. We examined differences in electrophysiological properties of the sino-atrial node (SAN) area between pediatric and adult patients with CHD.

**Methods:**

Epicardial mapping of the SAN was performed during sinus rhythm in 12 pediatric (0.6 [0.4–2.4] years) and 15 adult (47 [40–55] years) patients. Unipolar potentials were classified as single-, short or long double- and fractionated potentials. Unipolar voltage, relative R-to-S-amplitude ratio and duration of all potentials was calculated. Conduction velocity (CV) and the amount of conduction block (CB) was calculated.

**Results:**

SAN activity in pediatric patients was solely observed near the junction of the superior caval vein and the right atrium, while in adults SAN activity was observed even up to the middle part of the right atrium. Compared to pediatric patients, the SAN region of adults was characterized by lower CV, lower voltages, more CB and a higher degree of fractionation. At the earliest site of activation, single potentials from pediatrics consisted of broad monophasic S-waves with high amplitudes, while adults had smaller rS-potentials with longer duration which were more often fractionated.

**Conclusions:**

Compared to pediatric patients, adults with uncorrected CHD have more inhomogeneous conduction and variations in preferential SAN exit site, which are presumable caused by aging related remodeling. Long-term follow-up of these patients is essential to demonstrate whether these changes are related to development of SND and also atrial tachyarrhythmias early in life.

## Introduction

The population of patients with congenital heart disease (CHD) is increasing, as the survival of CHD patients has improved considerably due to advances in medical care early in life. However, with aging also the risk of developing atrial tachyarrhythmias such as atrial fibrillation (AF) increases (1). Furthermore, AF tends to develop in CHD patients at a younger age, resulting in greater mortality rates in this patient group (2). Sinus node dysfunction (SND) is also a common sequel in CHD patients and is in turn associated with heart failure and AF (3). SND encompasses disturbances in sino-atrial node (SAN) impulse generation and/or its propagation from SAN exit sites toward the atrial myocardium.

There is increasing evidence that genetic mutations which cause CHD are also associated with SND (4). Prior studies have indicated that abnormalities in anatomy and function of the SAN in patients with CHD are already present at birth predisposing them to development of SND relatively early in life (5). During aging, additional progressive structural remodeling of the right atrium (RA) of children with CHD may explain enhanced susceptibility to SND. In addition, progressive structural remodeling in the atria promotes inhomogeneous conduction which is associated with alterations in electrogram (EGM) morphology, such as fractionation and decreased peak-to-peak amplitudes.

Based on these observations, we therefore hypothesize that adult patients with CHD have more inhomogeneous conduction at the SAN area compared to pediatric patients. To confirm this hypothesis, we performed intra-operative high-resolution epicardial mapping of SAN activity in pediatric and adult patients with CHD undergoing primary surgical correction.

## Materials and Methods

### Study Population

Patients with CHD undergoing elective open-heart surgery were included in the present study. Exclusion criteria were hemodynamic instability, atrial pacing and previous cardiac surgery. The study was approved by the institutional medical ethical committee (MEC2015-373, MEC2019-0543) and written informed consent was obtained from all patients older than 16 years and from parents of children below the age of 16 years. The study was conducted according to the principles of the Declaration of Helsinki.

### Epicardial Mapping Procedure

**Figure 1:**
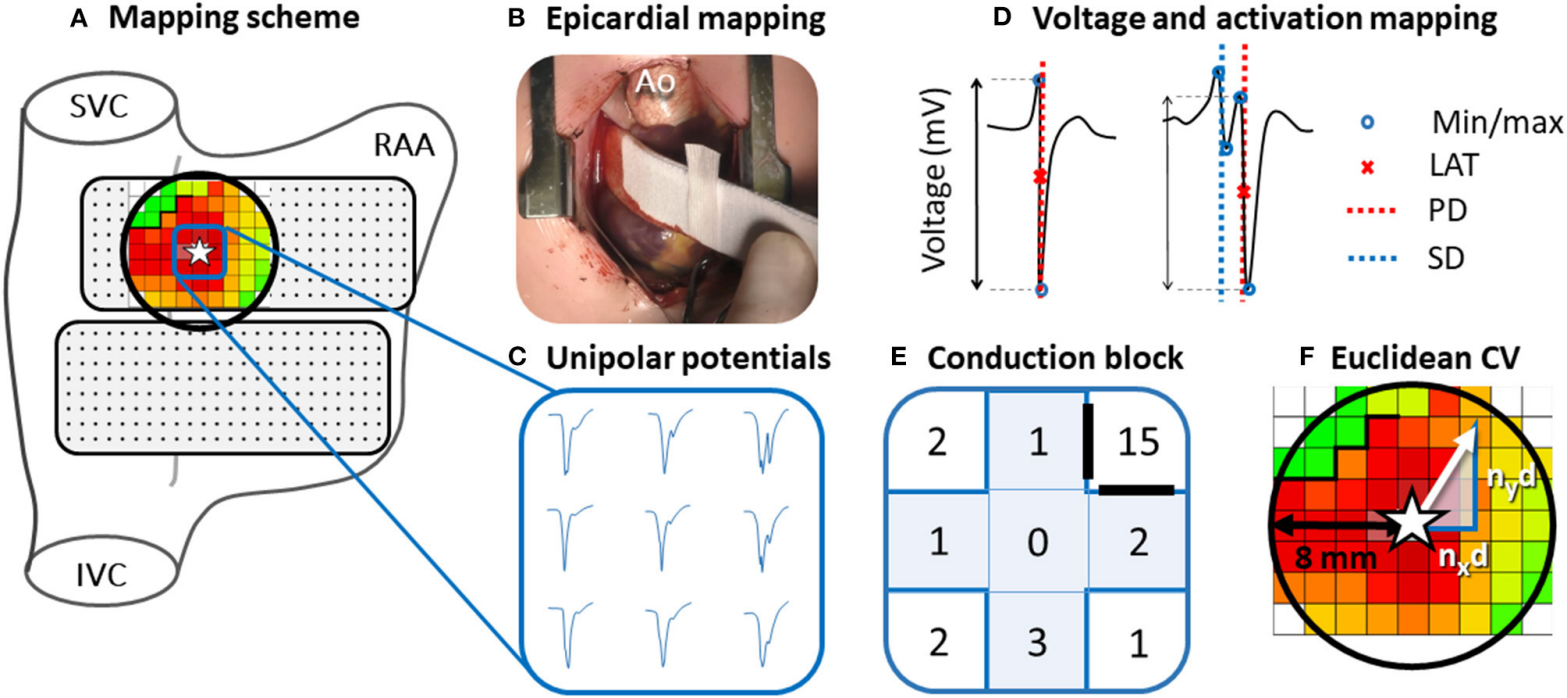
Epicardial mapping of the SAN. Projection of the 192-unipolar electrode array on a schematic posterior view of the right atrium **(A)** and intraoperative image of the mapping procedure **(B)**. An area with a radius of 8 mm around the SAN-FAP origin (star) is selected (black circle). Typical examples of nine unipolar potentials obtained at the center of the SAN-FAP are shown outside the mapping scheme **(C)**. These potentials are classified according to the number of deflections and potential voltage is determined as the peak-to-peak amplitude of the steepest (primary) deflection **(D)**. Five potentials directly surrounding the SAN-FAP origin (blue highlighted plus) are used to characterize potentials at the origin of SAN activation **(E)**. Conduction block is defined as a difference in local activation times (conduction time) between adjacent electrodes ≥12 ms. The Euclidean CV is analyzed by calculating the conduction times between the SAN-FAP origin and the neighboring electrodes in superior, caudal, lateral and medial direction divided by the distance *n*_*x*_*d* and *n*_*y*_*d* **(F)**. SVC, superior vena cava; IVC, inferior vena cava; RAA, right atrial appendage; Ao, aorta; LAT, local activation time; PD, primary deflection; SD, secondary deflection; CV, conduction velocity.

An overview of our data recording and processing approach is provided in Figure 1. Before institution of cardiopulmonary bypass, high-density (192 unipolar electrodes) and high-resolution (interelectrode distance 2 mm) epicardial mapping of SAN activity was performed (6). The indifferent electrode was connected to the subcutaneous tissue of the thoracic wall in the surgical field. Subsequently, a pacemaker wire, serving as a temporal reference electrode, was stitched to the superior lateral wall of the RA. Mapping of the superior RA started at the junction of the superior caval vein and RA, also covering the sulcus terminalis. Due to the large functional dynamic range of the SAN, the mapping array was subsequently moved to the middle and inferior part of the RA. Epicardial mapping at each location was performed for 5 seconds and included a calibration signal of 2 mV and 1,000 ms, a unipolar or bipolar reference EGM and all epicardial unipolar EGMs. Data were stored on a hard disk after amplification (gain 1,000), filtering (bandwidth 0.5–400 Hz), sampling (1 kHz) and analog to digital conversion (16 bits).

### Mapping Data Analysis

Mapping data were analyzed using custom-made software. Color-coded activation maps were constructed by annotating the steepest negative slope of atrial potentials recorded at every electrode, provided that the amplitude of an atrial deflection was at least two times the signal-to-noise ratio. In order to investigate conduction properties at different distances relative to the SAN focal activation pattern (SAN-FAP) origin, the Euclidean distances between the SAN-FAP origin and surrounding electrodes within a range of 8 mm were calculated (Figure 1). Consistent with prior intra-operative mapping studies, areas of CB were defined as interelectrode differences in local activation times (LATs) of ≥12 ms corresponding with an effective conduction velocity (CV) of <17 cm/s (right panel of Figure 1) (6). Because of the lack of any reference values, the same cut-off values were used for pediatric patients (5). The amount of CB is expressed as the number of interelectrode conduction times (CTs) ≥12 ms as a percentage of the total number of interelectrode CTs measured within 8 mm of the SAN-FAP.

### SAN Activation

All recordings were independently screened by two researchers based on the selection criteria for SAN-FAPs, as described previously (7). These criteria include: (1) The electrode from which the SAN-FAP originates (SAN-FAP origin) is the earliest site of activation in the atria, (2) in case of a neighboring electrode being simultaneously activated at the same time, this electrode should in turn be activated at least 2 ms earlier than its surrounding electrodes, and (3) the distance between the border of the mapping area and the SAN-FAP origin should be at least one electrode.

In case of stable, repetitive SAN activation patterns throughout the entire recording, the middle beat was included for analysis. Otherwise, one beat per unique SAN-FAP was included. If the SAN-FAP origin covered multiple electrodes with the same LATs, the electrode closest to the center of this earliest activated area was chosen as the SAN-FAP origin.

### Propagation Velocity of SAN-FAPs

CV within a radius of 8 mm around the origin of the SAN-FAP was analyzed by calculating the CTs between the SAN-FAP origin and the neighboring electrodes in superior, caudal, lateral and medial direction (Left panel Figure 1).


$$\begin{array}{l} CV=81\frac{\sqrt{{\left(n_{x}d\right)}^{2}+{\left(n_{y}d\right)}^{2}}}{t_{2}-t_{1}}\cdot 100\left[cm/s\right] \end{array}$$

With *n*_*x*_ and *n*_*y*_ the number of electrodes from the SAN-FAP origin in, respectively, the *x* and *y* direction, *d* the interelectrode distance (mm) and *t* the local activation time.

As there are multiple electrodes with the same distance to the SAN-FAP origin in the different directions, the median CVs from the origin of SAN-FAP toward the predefined distances within this area were calculated reflecting the overall SAN CV.

### Analysis of EGM Morphology

EGM morphology was automatically analyzed using custom-made software. As shown in the right lower panel of Figure 1, unipolar potentials were classified as single potentials (SP, one deflection), short double potentials (SDP, interval between deflections <15 ms), long double potentials (LDP, deflection interval ≥15 ms) or fractionated potentials (FP, ≥3 deflections). Fractionation delay is defined as the time difference between the first and last deflection of SDP, LDP and FP (8). From every potential, the peak-to-peak voltage and slope of the steepest deflection was measured. R/S-ratios of all SP were calculated by dividing the relative R- and S-wave amplitudes (9). Furthermore, duration of each SP within a radius of 8 mm around the origin of the SAN-FAP was calculated from the start to the end of the potential.

### Statistical Analysis

Normally distributed continuous variables were expressed as mean ± standard deviation and skewed variables as median values with interquartile ranges. Continuous data was analyzed using the Mann-Whitney U test or the Kruskal-Wallis H test. A *p*-value of <0.05 was considered statistically significant. Statistical testing was performed using Python.

## Results

### Pediatric Study Population

Twelve pediatric patients (median age 0.6 years [IQR 0.4–2.4], 4 females [33.3%]) scheduled for repair of ventricular septal defect (VSD) (*n* = 7, VSD in combination with atrial septal defect (ASD) type II (*n* = 5) or patent foramen ovale (*n* = 1)), ASD type II (*n* = 2), Tetralogy of Fallot with ASD type II (*n* = 1), subaortic stenosis (*n* = 1) and supravalvular aortic stenosis (*n* = 1) were included. RA dilatation was present in 2 patients. None of these patients had a history of atrial tachyarrhythmia, SND or heart failure.

### Adult Study Population

As shown in Table 1, a total of 15 adults were included (median age 47 years [IQR 40–55], 4 females [27%]) of whom 5 had a history of AF. Patients were scheduled for repair of partial anomalous venous return [*n* = 6, including repair of sinus venosus defect (*n* = 5)], ASD type II (*n* = 5), partial atrioventricular septal defect (*n* = 1), VSD (*n* = 2) and Ebstein anomaly (*n* = 1). RA dilatation was present in 9 patients and no patients had heart failure or SND. Characteristics of both pediatric and adult patients are summarized in Table 1.

**Table 1:** Patient characteristics.

	**Pediatric patients**	**Adult patients**
	***N =* 12**	***N =* 15**
Age (y)	0.6 [0.4–2.4]	46 ± 14
Female	4 (33.3%)	4 (27%)
BMI (kg/m^2^)	14.8 [13.9–16.4]	25.1 [22.8–35.1]
**Underlying congenital heart disease**		
ASD II	2	5
VSD	2	1
ASD II + VSD	4	–
Supravalvular AoS	1	–
Malalignment VSD + ASD II	1	–
ToF + ASD II	1	–
DSAS	1	–
PAVSD	–	1
PAPVR	–	1
SVD + PAPVR	–	5
Ebstein	–	1
DORV + VSD	–	1
**History of AF**		5
Paroxysmal AF	–	4
Persistent AF	–	1
**Cardiovascular risk factors**		
Hypertension	–	2
Hypercholesterolemia	–	4
**LVF**		
Good	12	12
Mild impairment	–	3
Right atrial dilatation	2	9

*Values are presented as N (%) or median [interquartile ranges]*.

*AF, Atrial fibrillation; AoS, Aortic stenosis; ASD, Atrial septal defect; BMI, Body mass index; DSAS, Discrete subaortic stenosis; LVF, Left ventricular function; PAPVR, Partial anomalous venous return; PAVSD, partial atrioventricular septal defect; SVD, Sinus venosus defect; ToF, Tetralogy of Fallot; VSD, ventricular septal defect*.

### Functional Dynamic Range of the SAN

**Figure 2:**
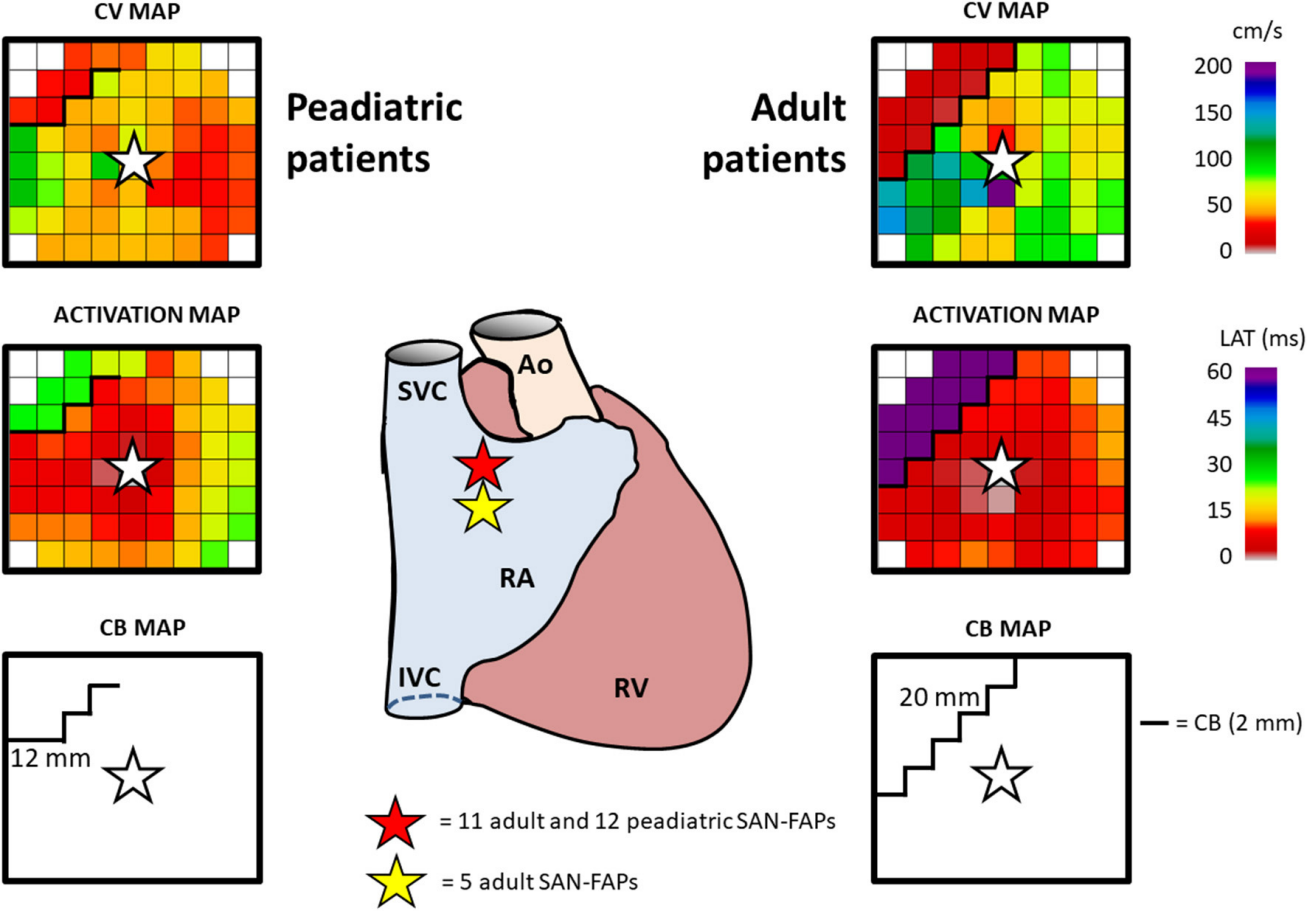
Typical example of SAN activation. The center panel demonstrates the location of the SAN-FAPs in both an adult and pediatric patient. Characteristics of SAN activation in a pediatric (left) and adult (right) patient include CV (top), activation map (middle) and CB (bottom). The SAN-FAP origin is indicated by a white star. SVC, superior vena cava; IVC, inferior vena cava; RA, right atrium; RAA, right atrial appendage; RV, right ventricle; Ao, aorta; CV, conduction velocity; CB, conduction block; SAN-FAP, sino-atrial node—focal activation pattern.

In one patient, who underwent cardiac surgery for a sinus venosus defect and partial anomalous pulmonary venous return, two different SAN exit sites were observed. All other patients had one SAN exit site giving rise to 28 SAN-FAPs. Repetitive SAN activity at the exact same site, as shown in Video 1, was observed in the whole population. As illustrated in Figure 2, SAN activity in pediatric patients was solely observed near the junction of the superior caval vein and RA. In contrast, in adults the functional dynamic range of the SAN was larger; SAN activity was observed from the superior caval vein-RA junction up to the middle part of the RA. Characteristics of all SAN-FAPs are specified in Table 2.

**Table 2:** Characteristics of SAN-FAPs.

	**Pediatric patients**	**Adult patients**	***p*** **-value**
Number of SAN-FAPs	12	16	–
Median CL (ms)	465 [421–491]	835 [754–873]	<0.001
**RA location**			
Superior	12	11	–
Middle	0	5	–
**Electrogram characteristics at the SAN-FAP region**				
Fractionation (%)	22.8 [18.8–29.8]	40.2 [25.0–45.3]	0.019
− SP	77.2 [70.2–81.2]	59.8 [54.7–75.0]	0.019
− SDP	10.5 [7.5–14.7]	14.1 [7.2–21.2]	0.289
− LDP	6.3 [3.8–10.4]	10.7 [5.3–20.3]	0.201
− FP	3.4 [1.3–5.3]	5.3 [0.0–14.1]	0.191
Voltage (mV)	5.2 [4.1–6.4]	2.6 [1.8–4.5]	0.004
− SP	6.4 [4.8–7.2]	3.4 [2.7–5.2]	0.010
− SDP	3.4 [2.3–4.0]	2.8 [1.6–3.8]	0.281
− LDP	2.1 [1.7–2.6]	1.2 [0.9–1.5]	0.041
− FP	1.2 [1.0–2.2]	0.9 [0.7–1.3]	0.435
Slope (V/s)	−2.04 [−2.31–−1.62]	−0.96 [−1.50–−0.53]	0.005
− SP	−1.14 [−1.36–−0.91]	−0.36 [−0.76–−0.24]	0.007
− SDP	−0.88 [−0.98–−0.77]	−0.61 [−0.93–−0.37]	0.055
− LDP	−0.56 [−0.69–−0.35]	−0.19 [−0.23–−0.14]	<0.001
− FP	−0.38 [−0.54–−0.37]	−0.24 [−0.40–−0.20]	0.118
Potential duration (ms)	40 [30–41]	61 [59–64]	<0.001
Fractionation delay (ms)	15 [12–18]	21 [12–24]	0.132
− SDP	8 [6–8]	6 [5–7]	0.265
− LDP	18 [17–24]	26 [20–31]	0.007
− FP	19 [15–26]	22 [18–31]	0.189
R/S ratio	0.91 [0.90–0.93]	0.90 [0.87–0.93]	0.381
CV (cm/s)	67.3 [62.1–74.5]	70.7 [63.9–75.0]	0.258
CB (%)	6.0 [4.9–7.6]	10.6 [7.7–13.1]	0.011
CB (mm)	12 [9,10,12,13]	20 [12–26]	0.030

*Values are presented as median [interquartile ranges] or incidence*.

*SAN-FAPs, Sino-atrial node—focal activation patterns; CL, cycle length; RA, Right atrium; SP, single potential; SDP, short double potential; LDP, long double potential; FP, fractionated potential; CV, conduction velocity; CB, conduction block*.

### Propagation Velocity of SAN-FAPs

Median CV within 4 electrodes (corresponding with a radius of 8 mm) from the origin of the SAN-FAPs did not significantly differ between adult and pediatric patients (70.7 cm/s [63.9–75.0] vs. 67.3 cm/s [62.1–74.5], *p* = 0.258). However, CV within 2 mm of the origin was significantly lower in adult patients (46.5 cm/s [29.9–56.4] vs. 25.4 cm/s [8.9–50.0], *p* = 0.011).

Figure 2 demonstrates typical differences in characteristics of SAN activation in a pediatric (left panel) and adult patient (right panel). Within an area of 201 mm^2^ (corresponding with a radius of 8 mm) around the origin of the SAN-FAP, significant more CB was observed in adults compared to pediatric patients (10.6 % [7.7–13.1] vs. 6.0 % [4.9–7.6], *p* = 0.011). In addition, lines of CB were also longer in adult patients (12 mm (9–13) vs. 20 mm (12–26), *p* = 0.030).

### Characteristics of Unipolar Potentials at the SAN-FAP Region

Compared to pediatric patients, there were less SPs in adult patients (59.8 % [54.7–75.0] vs. 77.2 % [70.2–81.2], *p* = 0.019); although there were no significant differences in the prevalence of SDPs (10.5 % [7.5–14.7] vs. 14.1 % [7.2–21.2], *p* = 0.289), LDPs (6.3 % [3.8–10.4] vs. 10.7 % [5.3–20.3], *p* = 0.201) and FPs (3.4 % [1.3–5.3] vs. 5.3 % [0.0–14.1], *p* = 0.191).

SPs in the adult population had lower voltages (3.4 mV [2.7–5.2] vs. 6.4 mV [4.8–7.2], *p* = 0.010) and were less steep (−0.36 V/s [−0.76 – −0.24] vs. −1.14 V/s [−1.36 – −0.91], *p* = 0.007), while the R/S ratio was comparable (0.90 [0.87–0.93] vs. 0.91 [0.90–0.93], *p* = 0.381). Also, the potential duration of SPs was longer in adult patients compared to pediatric patients (61 ms [59–64] vs. 40 ms [30–41], *p* < 0.001).

SDPs, LDPs and FPs were present in respectively 75%, 92% and 92% of the pediatric and in 63%, 88% and 81% of the adult population. SDPs and FPs in pediatric patients did not differ from adult patients in voltage, slope or duration (all p>0.055). Compared to adult patients, LDPs obtained from pediatric patients had lower voltages (1.2 mV [0.9–1.5] vs. 2.1 mV [1.7–2.6], *p* = 0.041) and less steep potential slopes (−0.19 V/s [−0.23 to −0.14] vs. −0.56 V/s [−0.69 to −0.35], *p* < 0.001). The delay of only LDPs was longer in adult patients (26 ms (20–31) vs. 18 ms (15–24), *p* = 0.007).

### Characteristics of Unipolar Potentials at the SAN-FAP Origin

**Figure 3:**
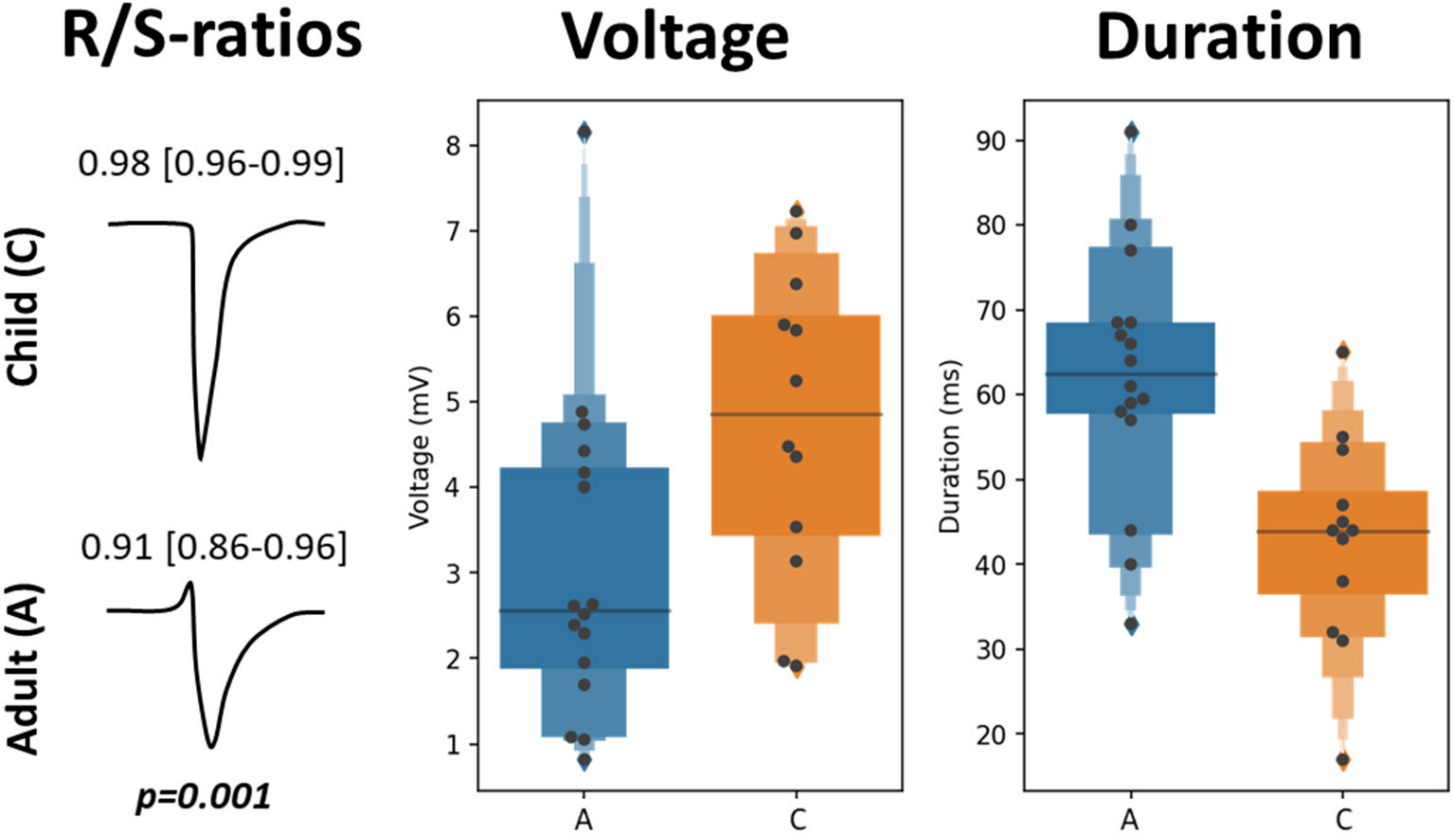
EGM morphology characteristics at the SAN-FAP origin. Two typical examples of EGM morphologies at the SAN-FAP origin obtained from a pediatric (top) and adult patient (bottom), and the corresponding R/S ratios (left panel). The SP voltage (center) and duration (right) distributions obtained from the adult (blue) and pediatric (orange) population are shown in the boxplots.

Characteristics of EGM morphology at the origin of the SAN-FAP are summarized in Table 3 and illustrated in Figure 3. The left panel shows two typical examples of EGMs obtained from a pediatric and adult patient. Potentials recorded from pediatric patients consisted of broad monophasic S-waves with high amplitudes (5.1 mV [3.4–6.0]) and R/S ratios close to 1 (R/S ratio 0.98 [0.96–1.00]). In contrast, as shown in the left lower panel, the origin potentials in adults contained smaller S-wave amplitudes (2.5 mV [1.9–3.9], *p* = 0.007), larger R-wave amplitudes (0.17 mV [0.06–0.45], *p* = 0.036) and smaller R/S ratios (0.92 [0.87–0.96], *p* < 0.001). The middle panel of Figure 3 shows that the origin potential peak-to-peak amplitudes were also lower in adult compared pediatric patients (2.6 mV [1.9–4.2] and 4.9 mV [3.4–6.0], *p* = 0.014). SP duration, as shown in the right panel of Figure 3, was substantially longer (45 ms [37–50] vs. 66 ms [60-70], *p* < 0.001) and the slope was less steep in adults (−1.4 V/s [−1.9 to −0.9] vs. −0.6 V/s [−1.1 to −0.3], *p* = 0.015). At the SAN-FAP origin, SDP and LDPs were found in, respectively, 33% of the pediatric and 69% of the adult patients whereas FPs were only found in adult patients (25%).

**Table 3:** Characteristics of SAN-FAPs origin.

	**Pediatric patients**	**Adult patients**	***p*** **-value**
Voltage (mV)	4.9 [3.4–6.0]	2.6 [1.9–4.2]	0.014
R/S ratio	0.98 [0.96–1.00]	0.92 [0.87–0.96]	<0.001
R-wave (mV)	0.10 [0.00–0.13]	0.17 [0.06–0.45]	0.036
S-wave (mV)	5.06 [3.35–5.96]	2.49 [1.85–3.90]	0.007
Slope (V/s)	−0.65 [−0.84–−0.35]	−0.30 [−0.55–−0.15]	0.030
Potential duration (ms)	45 [37–50]	66 [60–70]	<0.001
R-wave duration (ms)	1 [0–3]	6 [5–12]	0.003
S-wave duration (ms)	41 [36–45]	56 [53–58]	<0.001

*Values are presented as median [interquartile ranges]*.

*SAN-FAPs, Sino-atrial node—focal activation patterns*.

### Impact of AF Episodes

Supplementary Table 1 demonstrates the differences in electrophysiological parameters at the SAN-FAP region between adult patients with and without history of AF. In patients with AF, CV was slower than in patients without AF (45.2 cm/s [35.1–63.9] vs. 73.3 cm/s [70.7–75.0], *p* = 0.039), though there was a similar amount of CB (11.7 % [10.2–18.1] vs. 9.0 % [7.5–12.7], *p* = 0.182). In addition, SPs recorded from the SAN area in patients with AF had smaller peak-to-peak voltages (4.1 mV [3.2–5.5] vs. 2.2 mV ([2.0–3.1], *p* = 0.027) and less negative slopes (−0.52 V/s [−1.08 to −0.36] vs. −0.24 V/s [−0.26 to −0.16], *p* = 0.012).

## Discussion

### Key Findings

Compared to pediatric patients, the SAN region of adult patients with CHD is characterized by slower conduction, lower SP and LDP voltages, more areas of CB and a higher degree of potential fractionation. Also at the origin of the SAN-FAP, we observed lower SP voltages in combination with less steep slopes and prolonged SP duration in adult patients. Solely in adult patients, we also observed SAN activation at the middle part of the RA, which may indicate changes in preferential SAN exit site due to chronic volume overload induced structural remodeling. Patients with a history of AF had even slower conduction and lower voltages, which may indicate disturbances in SAN impulse generation and its propagation toward the atrial myocardium.

### Mapping of Human Sino-Atrial Node Activation

This is the first epicardial mapping study on SAN activation in CHD patients and comparative data is therefore lacking. So far, only a few *ex-vivo* and *in-vivo* SAN mapping studies have been performed in non-CHD patients (7, 10–12). Despite the SAN is limited to an area of ≈1–2cm, its functional dynamic range may cover the whole area between the superior and inferior caval vein. Recently, Fedorov et al. demonstrated the presence of two spatially distinct dominant pacemaker sites at the superior and inferior RA by performing optical *ex-vivo* human SAN mapping followed by histological examination (12). Furthermore, our research group recently performed simultaneous endo-epicardial mapping of *in-vivo* SAN activation in non-CHD patients and found inter-individual differences in SAN exit-pathways covering a large area at the RA (7).

Previous endocardial mapping studies showed spatial caudal shifting of SAN activity in patients with heart failure, SND and a history of AF (7, 10, 11). Structural remodeling and consequently endo-epicardial conduction disorders may block propagation of wavefronts from the superior SAN exit sites thereby shifting SAN activity toward inferior sites. We observed changes in electrophysiological properties at the surrounding of the SAN in adults compared to pediatrics with CHD, which support the assumption that adults with uncorrected CHD have a relevant higher degree of structural remodeling in the RA due to long-standing volume overload.

### Electrophysiological Alterations Surrounding the SAN

Electrophysiological differences in SAN activation between pediatric and adult patients may partly be elucidated by differences in the 3-dimensional anatomy of the SAN. Histological examination of the SAN in 10 infants and 11 adults indeed showed remarkable differences in SAN geometry (adult SAN length: 13.8 ± 2.3 mm, width: 3.4 ± 0.5 mm; pediatric SAN length: 5.1 ± 0.7 mm, width: 0.9 ± 0.2 mm) (13). In addition, the SAN was more superficial in pediatric patients with a mean distance to the epicardium of 0.1 mm compared to 0.3 mm in adults. Hence, a more compact functional dynamic range of the SAN in pediatrics may be explained by a smaller SAN with none or underdeveloped SAN exit sites.

EGMs recorded at the SAN-FAP most likely consist of a monophasic S-wave morphology. In pediatric patients, in whom the SAN is more superficially located and is surrounded by less fibrofatty tissue, we indeed observed more monophasic S-wave morphology at the origin (upper panel of Figure 3). However, in adults, in whom the SAN is embedded in deeper layers of the atrial wall and in whom the SAN exit pathways are surrounded by more fibrofatty tissue, the sinus rhythm wavefront has to propagate toward the surface before it can spread across the remainder of the epicardium, resulting in a small R-wave preceding a large S-wave (lower panel of Figure 3) (9). We postulate that in adult CHD patients with chronic RA volume overload and hence more advanced 3-dimensional structural remodeling, this would cause even larger relative R-wave amplitudes due to the more intramural location. Enhanced structural remodeling in the SAN exit-pathways may cause prolongation of potential duration and slowing of atrial conduction, which is consistent with our findings.

### Interrelationship Between SND and Atrial Tachyarrhythmia

SND in patients with CHD is a frequent indication for pacemaker implantation and may even result in sudden cardiac death (3, 14). In patients with CHD, aging itself and complex surgery at the atrial level (e.g., Mustard and Senning procedure) have been associated with a higher incidence of SND (15, 16). However, there is also literature indicating that SND in this population is not related to the post-operative course only. First, several studies have reported SND in patients with CHD already before surgical correction (17–19). Second, reports on SND in fetuses with CHD suggest that abnormalities in the 3-dimensonal structure or function of the SAN may also predispose these patients to SND (20). SND may be a consequence of electrical and structural remodeling induced by tachyarrhythmia, as multiple studies have shown that AF may cause SND. On the other hand, there is also enough evidence illustrating that a substantial amount of patients with AF does not develop SND or is diagnosed with SND prior to AF (21).

### Study Limitations

Due to lack of histology it might be possible that some SAN-FAPs were not caused by SAN activity, but by ectopic focal discharges in the RA wall. However, because during stable sinus rhythm all SAN-FAPs were repetitive and solely found in the superior and mid RA, this is very unlikely. The SAN recovery time (SNRT) and sino-atrial conduction time (SACT) should preferably be examined by pacing and extra stimulation to estimate SAN automaticity and conduction. However, due to limited time during surgery, this was not possible. Whether general anesthesia has a significant effect on the SAN and whether this effect is unequally distributed between adult and pediatric patients has yet to be investigated. Sharpe et al. showed that propofol has no direct effect on SAN function (22). Also a standard anesthetic protocol was used for all patients, therefore equal dispersion of possible effect can be assumed. An inevitable effect of *in-vivo* mapping is lack of histology and intramural SAN analyses. Due to the invasive nature of our epicardial mapping approach, we do not have epicardial mapping data of patients without structural heart disease at our disposal for comparison.

## Conclusion

Compared to pediatric patients, adults with uncorrected CHD have more inhomogeneous conduction at the SAN area. Also, variations in preferential SAN exit site were solely observed in adult patients. These observations indicate that adult patients with uncorrected CHD have more aging related remodeling which may partly explain why this population is prone to develop SND and atrial tachyarrhythmias. Long-term follow up of this population is essential to demonstrate whether these changes are indeed related to development of atrial tachyarrhythmias early in life.

## Data Availability Statement

The datasets presented in this article are not readily available because of EU privacy law. Requests to access the datasets should be directed to NG, n.m.s.degroot@erasmusmc.nl.

## Ethics Statement

The studies involving human participants were reviewed and approved by METC Erasmus MC. Written informed consent to participate in this study was provided by the participants' legal guardian/next of kin.

## Author Contributions

RK and MS contributed to data acquisition and analysis, manuscript drafting and conceptual thinking. NR, FW, RZ, WL, PW, PJ, AB, and YT contributed to data acquisition and critically revising the manuscript. NG contributed to manuscript drafting and conceptual thinking. All authors contributed to the article and approved the submitted version.

## Funding

NG was supported by funding grants from CVON-AFFIP [Grant Number 914728], NWO-Vidi [Grant Number 91717339], Biosense Webster USA [ICD 783454] and Medical Delta.

## Conflict of Interest

The authors declare that the research was conducted in the absence of any commercial or financial relationships that could be construed as a potential conflict of interest.

## Publisher's Note

All claims expressed in this article are solely those of the authors and do not necessarily represent those of their affiliated organizations, or those of the publisher, the editors and the reviewers. Any product that may be evaluated in this article, or claim that may be made by its manufacturer, is not guaranteed or endorsed by the publisher.

## Abbreviations

AF: Atrial fibrillation
ASD: Atrial septal defect
CB: Conduction block
CHD: Congenital heart disease
CT: Conduction time
CV: Conduction velocity
EGM: Electrogram
LAT: Local activation time
LVF: Left ventricular function
LVEF: Left ventricular ejection fraction
RA: Right atrium
SAN: Sino-atrial node
SAN-FAP: Sino-atrial focal activation pattern
SND: Sinus node dysfunction
VSD: Ventricular septal defectAbstract.
