# Supplementary material for: In-vivo Sino-Atrial Node Mapping in Children and Adults With Congenital Heart Disease
Source: Front Pediatr. 2022 Jul 1;10:896825. doi: 10.3389/fped.2022.896825 (PMC9283725; doi:10.3389/fped.2022.896825)
Supplement: Supplementary file 1 [file Table_1.DOCX]

**Supplemental Table 1. Characteristics of SAN-FAPs between patients with and without AF**

|  | **Without AF history**  **N= 10** | **With history of AF**  **N= 5** | **p-value** |
| --- | --- | --- | --- |
| Fractionation (%) | 40.0 [28.7–41.0] | 50.0 [14.0–79.1] | 0.248 |
| - SP | 60.0 [59.0–71.3] | 50.0 [20.9–86.0] | 0.248 |
| - SDP | 16.2 [9.5–21.0] | 3.5 [0.0–20.3] | 0.182 |
| - LDP | 12.7 [5.3–20.3] | 10.5 [6.2–10.9] | 0.455 |
| - FP | 5.3 [0.0–8.4] | 18.8 [0.0–25.6] | 0.147 |
| Voltage (mV) | 3.5 [2.3–4.6] | 1.9 [1.5–2.5] | 0.106 |
| - SP | 4.1 [3.2–5.5] | 2.2 [2.0–3.1] | 0.027 |
| - SDP | 2.9 [1.9–4.0] | 1.8 [1.6–2.3] | 0.325 |
| - LDP | 1.2 [0.9–1.5] | 1.2 [0.7–1.6] | 0.500 |
| - FP | 0.7 [0.6–1.0] | 1.3 [1.1–1.3] | 0.129 |
| Slope (V/s) | -0.53 [-0.73–-0.35] | -0.24 [-0.25–-0.19] | 0.027 |
| - SP | -0.52 [-1.08–-0.36] | -0.24 [-0.26–-0.16] | 0.012 |
| - SDP | -0.70 [-0.90–-0.37] | -0.53 [-0.83–-0.35] | 0.087 |
| - LDP | -0.19 [-0.23–-0.17] | -0.17 [-0.22–-0.14] | 0.325 |
| - FP | -0.25 [-0.38–-0.18] | -0.24 [-0.38–-0.24] | 0.367 |
| Potential duration (ms) | 62 [62–65] | 57 [56–63] | 0.095 |
| Fractionation delay (ms) | 21 [8–26] | 26 [16–36] | 0.070 |
| - SDP | 6 [5–6] | 9 [7–10] | 0.044 |
| - LDP | 24 [20–31] | 32 [26–35] | 0.214 |
| - FP | 23 [17–30] | 18 [18–29] | 0.289 |
| R/S ratio | 0.90 [0.86–0.94] | 0.90 [0.89–0.91] | 0.367 |
| CV (cm/s) | 73.3 [70.7–75.0] | 45.2 [35.1–63.9] | 0.039 |
| CB (%) | 9.01 [7.54–12.70] | 11.71 [10.20–18.06] | 0.182 |
| CB (mm) | 18 [11–22] | 26 [20–26] | 0.139 |

Values are presented as median [interquartile ranges].

**AF**= Atrial fibrillation, **SP**= single potential, **SDP**= short double potential, **LDP**= long double potential, **FP**= fractionated potential, **CV**= conduction velocity, **CB**= conduction block
